# Implication of Sialidases in *Salmonella* Infection: Genome Release of Sialidase Knockout Strains from *Salmonella enterica* Serovar Typhimurium LT2

**DOI:** 10.1128/genomeA.00341-17

**Published:** 2017-05-11

**Authors:** Narine Arabyan, Allison M. Weis, Bihua C. Huang, Bart C. Weimer

**Affiliations:** a Department of Population Health and Reproduction, School of Veterinary Medicine, UC Davis, Davis, California, USA; b 100K Pathogen Genome Project, UC Davis, Davis, California, USA

## Abstract

Sialidases, which are widely distributed in nature, cleave the α-ketosidic bond of terminal sialic acid residue. These emerging virulence factors degrade the host glycan. We report here the release of seven sialidase and one sialic acid transporter deletion in *Salmonella enterica* serovar Typhimurium strain LT2, which are important in cellular invasion during infection.

## GENOME ANNOUNCEMENT

Sialidases are widely distributed among microbes and are one of the least characterized and ill-defined glycosyl hydrolases. Sialidases have been associated with several diseases. Sialidases play a critical role in microbiology by mediating metabolism, adherence, and infection, and they are important regulators of alternate complement pathway activation, red blood cell destruction, cell growth, cell adhesion, and tumor metastasis in mammalian systems (1–5). Recently, the importance of sialidases in infection and commensalism has come to light, opening the potential to use newly measured genomic diversity as a means to investigate infection mechanisms. Though antibiotics are available for treatment of bacterial infections, inhibitors of all sialidases and new drug targets may be medically useful where sialidase activity has been correlated with severe infection pathology.

The presence of sialidases is highly correlated with the progress and severity of the disease, and the most probable role of sialidases is for successful attachment and colonization. Microbes use sialidases to reveal the cell surface that holds sialic acid-containing cell membrane receptors during infection. Sialidases play an important role in infection by altering the host glycan structure to gain access of the host epithelial cells by binding to terminal sialic acid receptors to initiate glycan degradation (6). The two sialidases (*ΔnanH* and* ΔSTM1252*) from *Salmonella enterica* serovar Typhimurium LT2 have the same domains and function as sialidases, but they are structurally very different, indicating domain shuffling and lack of structural conservation; therefore, this difference led to different invasion phenotypes during the *in vitro* infection of differentiated colonic epithelial cells (Caco-2) (6).

The 100K Pathogen Genome Project (http://www.100kgenomes.org) is a large-scale sequencing consortium that offers the use of new next-generation sequencing methods to provide cutting-edge methods for pathogen detection and control in the food supply. This project is focused on producing genomes of pathogenic isolates from the environment, plants, animals, and humans worldwide, providing new insights into the genetic diversity of *Salmonella* spp. and other foodborne pathogens. These seven sialidase and one sialic acid transporter mutant strain were constructed in the Weimer Laboratory (UC Davis, Davis, CA, USA) (6) as described by Datsenko and Wanner (7). Cultures were grown on 1.5% Luria–Bertani agar (Difco, Franklin Lakes, NJ, USA), with 10 µg/mL of chloramphenicol at 37°C, and then lysed (8). Genomic DNA was extracted (9), checked for quality (10), and fragmented (11). The 350- to 500-bp libraries (12, 13) were indexed (96 genomes/lane) and sequenced (Illumina HiSeq 3000; 150-bp paired-end) (14–16) at the UC Davis DNA Technologies Core. Paired-end reads were *de novo* assembled using CLC Workbench version 6 with default parameters. Here, the 100K Pathogen Genome Project has assembled seven genomes of single and double sialidases and one sialic acid transporter deletion strain of S. Typhimurium LT2.

### Accession number(s).

All sequences are publicly available and can be found at the 100K Pathogen Genome Project (NCBI PRJNA186441) in the Sequence Read Archive (http://www.ncbi.nlm.nih.gov/sra); genome assemblies can be found in NCBI GenBank (Table 1).

**TABLE 1:** *S*. Typhimurium LT2 sialidase and sialic acid transporter deletion mutants

GenBank accession no.	SRA accession no.	Isolate name	Gene deleted	No. of contigs	Coverage (×)	Total genome size (bp)	No. of coding sequences
MWQQ00000000	SRR5279339	BCW_7500	*ΔnanT*	65	143	4,895,101	4,810
MWVC00000000	SRR5288771	BCW_7514	*ΔinvA::ΔnanH*	240	15	4,892,397	4,898
MWQR00000000	SRR5279338	BCW_7515	*ΔinvA::ΔSTM1252*	74	130	4,892,686	4,819
MWQS00000000	SRR5279337	BCW_7516	*ΔmelA::ΔnanH*	72	142	4,870,638	4,785
MWQT00000000	SRR5279336	BCW_7517	*ΔmelA::ΔSTM1252*	59	302	4,895,400	4,805
MWQU00000000	SRR5279335	BCW_7518	*ΔnanH::ΔSTM1252*	54	106	4,893,964	4,803
MWQV00000000	SRR3622954	BCW_8441	*ΔSTM1252*	57	165	4,894,714	4,806
MWQW00000000	SRR3622955	BCW_8442	*ΔnanH*	60	139	4,894,435	4,815
